# Individual and Environmental Correlates of Adolescents’ Moral Decision-Making in Moral Dilemmas

**DOI:** 10.3389/fpsyg.2021.770891

**Published:** 2021-11-24

**Authors:** Dario Bacchini, Grazia De Angelis, Mirella Dragone, Concetta Esposito, Gaetana Affuso

**Affiliations:** ^1^Department of Humanities, University of Study of Naples Federico II, Naples, Italy; ^2^Department of Psychology, University of Campania “Luigi Vanvitelli”, Caserta, Italy

**Keywords:** moral judgment, trolley problem, utilitarian vs. deontological judgment, moral disengagement, community violence, values, callous-unemotional traits, parental rejection

## Abstract

While extensive research has been conducted on adults’ judgments in moral sacrificial dilemmas, there is little research on adolescents. The present study aimed at: (1) adding further empirical evidence about adolescents’ moral decisions (deontological vs. utilitarian) in sacrificial moral dilemmas and (2) investigating how these moral decisions relate with gender, school grade, emotional traits (callous-unemotional traits), context-related experiences (perceived parental rejection and community violence exposure), and moral-related factors (moral disengagement and universalism value). A sample of 755 Italian adolescents (54.7% females; Mean age=16.45, *SD=*1.61) attending the second and the fifth year of secondary school took part in the study. Two sacrificial trolley-type dilemmas (where harmful actions promote the greater good) were presented. In the “switch” scenario (impersonal sacrificial dilemma), the choice is whether to hit a switch to save five people killing only one person. In the “footbridge” scenario (personal sacrificial dilemma), the choice is whether to push a large man off a footbridge saving five persons. For each scenario, participants had to indicate whether the proposed action was “morally acceptable” or not. Data were analyzed performing generalized linear mixed models. Our results showed that: (1) Adolescents were more likely to indicate as admissible to hit the switch rather than to push the large man; (2) male adolescents, compared to females, were more likely to say it was morally acceptable to intervene in the footbridge dilemma, whereas younger adolescents said it was morally acceptable both in the switch and the footbridge situations; and (3) higher levels of callous-unemotional traits, perceived parental rejection, and moral disengagement, on the one hand, and lower levels of universalism, on the other hand, were associated to higher admissibility to intervene in the footbridge scenario. Higher community violence exposure was associated with a lower propensity to intervene in the switch scenario. Overall, the present study expands the research on sacrificial dilemmas involving a sample of adolescents. The findings support previous studies concerning the role of emotions in making moral decisions but, at the same, open new perspectives regarding the role of contextual experiences and moral-related factors.

## Introduction

In the last two decades, psychological research on morality assigned a prominent role to the intuitionist models, according to which moral judgments are driven by automatic, unconscious, and affective reactions (Haidt, 2001; Cosmides and Tooby, 2004; Cushman et al., 2006; Hauser et al., 2007; Haidt, 2012) rather than by conscious cognitive-based processes (Kohlberg, 1968; Turiel, 1989; Turiel, 2006).

From the sixties to the 2000s, psychological research on moral development has yielded a wealth of evidence in support of the assumption that reasoning is the milestone of moral judgment. In Kohlberg’s (1969) theory, morality is believed to evolve toward increasingly advanced stages accordingly to cognitive development. Social domain theorists (Turiel, 1989; Smetana, 2006, 2013) emphasized the capability of children to form judgments entailing the distinction between a moral domain, focused on impersonal and compulsory rules pertaining to welfare, justice, and rights, and a conventional domain, pertaining to not generalizable, arbitrary, shared social rules. From a different perspective, within the framework of the social cognitive theory, Bandura (1986) introduced the concept of moral agency according to which the conscious acceptance of moral standards guides individuals’ beliefs and behaviors. Similarly, the theory of values (Schwartz, 1992) claimed that values are enduring goals that refer to “what people consider important” (Roccas et al., 2002) and identified ten universal values, among which “universalism,” in particular, represents intrinsically moral goals guiding individuals’ moral evaluations (Schwartz, 2007).

Since 2000, the assumption that moral judgment is founded on rational adherence to moral principles has been strongly challenged. An increasing number of theorists proposed an alternative approach – embedded within the evolutionary paradigm – according to which the human mind is pre-programmed to automatically react to social cues implying moral decisions. Intuitionist approaches minimized the role of cognitive processes in making moral judgments, emphasizing, on the contrary, the role of automatic and innate mechanisms. In this perspective, the role of reasoning is reduced to producing justifications, post hoc rationalizations following a pre-existing judgment (Haidt, 2001). This change of paradigm represents a challenge when shifting toward a developmental perspective, as the emphasis on innate mechanisms dramatically overshadows the role of the development. As moral decisions are triggered by automatic responses, differences between children and adults in making moral decisions seem to disappear.

In early 2000, the dual-process theory (Greene et al., 2001; Greene, 2007) proposed a synthesis between the recent intuitionist models and the more traditional approaches to morality, suggesting that both intuitive emotional responses and more controlled cognitive responses play a crucial role in moral judgment. More specifically, while emotional processes were identified as the basis for deontological judgments, cognitive processes were considered the basis of utilitarian judgments. Consistently, the so-called trolley problem, introduced in late `60s just to investigate the processes underlying utilitarian vs. deontological moral judgments, became one of the most widely used tools in the research in this theoretical framework. Utilitarian judgments can be defined as judgments endorsing actions (even harmful) that promote the greater good (Greene, 2007) and as judgments that privilege aggregate welfare over that of a small number of individuals. Deontological judgments, on the other hand, are based on an immediate feeling that a specific action could be intrinsically “wrong,” irrespective of its consequences. The trolley problem and its variants (see later for a detailed description) are a prototypical dilemma where individuals have to choose whether it is permissible to sacrifice one life to save five others. Individuals answering “no” think that killing a person is intrinsically wrong, irrespective of its consequences and thus not acceptable, even if it would save the lives of several people. Individuals answering “yes” think that the consequences of any action are the focus and, thus, that sacrificing the welfare of one person can be considered right if it leads to saving the lives of several people. The first one is considered a “deontological response,” in which the emphasis is on moral rules, most often articulated in terms of rights and duties; the second one is considered a “utilitarian response,” in which the emphasis is on the consequences, more specifically, on maximizing benefits for the largest number of people. From a rationalist perspective, utilitarian and deontological choices would express an individual’s personal philosophical perspective. Nonetheless, extensive research has provided evidence that individuals make their judgments based on specific triggers which are present in the proposed dilemmas. More specifically, the dual-process theory (Greene et al., 2001; Greene, 2007) posits that when an impersonal action is required to save five human lives sacrificing the life of one, most individuals tend to make a utilitarian choice, whereas when a personal action is required most individuals tend to make a deontological choice, beyond their personal beliefs and reasoning.

In the present research, we aimed to increase the knowledge in this field of study in two ways. Firstly, investigating adolescents’ responses to trolley dilemma (and its variants) since, with few exceptions (e.g., Dahl et al., 2018), only a few studies involving individuals in the developmental age have been conducted so far. Secondly, analyzing the concurrent contribution of emotional (i.e., callous-unemotional traits), contextual (i.e., family and neighborhood), and moral-related (i.e., moral disengagement and moral values) variables to deontological vs. utilitarian judgments. Many studies investigated how emotions affected the responses to the trolley dilemma and its variants, but the samples they used included only adults. Furthermore, only a few studies have taken into account the role of family and we are not aware of any study investigating the role of neighborhood and moral cognitions with respect to the tendency to make utilitarian vs. deontological choices.

### Utilitarian Vs. Deontological Approach: Individuals’ Responses to Sacrificial Moral Dilemmas and Their Correlates

In Foot (1967) proposed a dilemma in which: “A runaway trolley is headed for five people who will be killed if it proceeds on its present course. The only way to save these people is to hit a switch that will turn the trolley onto a sidetrack, where it will run over and kill one person instead of five” (Greene, 2007, pp. 41–42). This is the text of the well-known trolley dilemma, of which many variants have been proposed in the following years. One of the most famous variants was the footbridge case (Thomson, 1985), in which: “A runaway trolley threatens to kill five people, but this time you are standing next to a large stranger on a footbridge spanning the tracks, in between the oncoming trolley and the five people. The only way to save the five people is to push this stranger off the bridge and onto the tracks below. He will die as a result, but his body will stop the trolley from reaching the others” (Greene, 2007, p. 42). Respondents had to choose if it was okay to turn the trolley, in the trolley case, or push the large man onto the tracks, in the footbridge case, “in order to save five people at the expense of one” (Greene, 2007, pp. 41–42). The interesting evidence emerging from a large literature was that, while people primarily consider acceptable turning the trolley, on the contrary, they primarily consider it unacceptable pushing the large man onto the tracks (see Awad et al., 2020). In other words, differently from what it would be expected on the basis of the rationalist perspective, while people primarily exhibit a utilitarian response to the standard trolley case, they primarily exhibit a deontological response to the footbridge case. Greene and colleagues’ dual-process model (Greene et al., 2001; Greene, 2007) provides an explanation of this difference positing that, depending on the characteristics of the situation, cognitive-driven or emotion-driven processes are primarily activated. More specifically, the footbridge case describes an “up-close and personal” (Greene, 2007, p. 43) situation, since the large man has to be personally pushed onto the tracks to stop the trolley, while the trolley situation, although bringing to the same consequence (the death of a person), requires just an impersonal action like hitting a switch. Therefore, the footbridge case turns out to be more morally salient and tends to evoke a dominant negative emotional response, leading to a primarily deontological response. On the contrary, the trolley situation, which is not associated with this dominant emotional response, allows more pragmatic cost-benefit analysis (Greene et al., 2001, 2004; Greene, 2007), leading to a primarily utilitarian response. Many studies investigating the link between emotional reactions to dilemmas and moral judgment supported the basic assumptions of the dual-process model (see Greene, 2014). Individuals with brain injuries altering affective reactions or individuals with a low level of affective empathy made more utilitarian judgments in sacrificial moral dilemmas (Koleva et al., 2014; Patil and Silani, 2014; Takamatsu and Takai, 2019; Dinić et al., 2021). Conversely, the examination of the role of cognitive empathy so far has provided controversial results: While some studies evaluating responses to moral everyday dilemmas evidenced a reduced tendency to make utilitarian choices in individuals with lower levels of cognitive empathy (Takamatsu, 2019), other researchers found that in sacrificial moral dilemmas the tendency to make utilitarian judgments is associated with even a selective impairment of cognitive empathy (Gleichgerrcht et al., 2013; Bacchini et al., 2018).

Other studies have focused on other possible correlates of the utilitarian choice, starting from the dimensions expected to be related to an empathic deficit, as some personality traits, like psychopathic traits and, more generally, Dark Triad traits. Research about psychopathy has evidenced that both incarcerated, clinical psychopaths (Koenigs et al., 2012; Rosas and Koenigs, 2014) and non-incarcerated, subclinical individuals with psychopathic tendencies show a preference for utilitarian solutions on emotionally aversive moral dilemmas (Glenn et al., 2010; Bartels and Pizarro, 2011; Langdon and Delmas, 2012; Gao and Tang, 2013; Djeriouat and Trémolière, 2014; Kahane et al., 2015; Patil, 2015; Balash and Falkenbach, 2018) confirming that emotionally callous personalities are more prone to endorse utilitarian judgment. However, other studies failed to find significant associations (Glenn et al., 2009; Cima et al., 2010; Pujol et al., 2012) or showed less consistent results (Gao and Tang, 2013). However, the association between dark triad traits and utilitarian judgment seems to be reduced when other personality traits, such as Honesty/Humility (Dinić et al., 2021) or moral foundations, such as Care/Harm (Djeriouat and Trémolière, 2014) were controlled. The studies evidencing the negative relation between utilitarian responses to sacrificial moral dilemmas and the endorsement of moral foundations (Koleva et al., 2014) suggested the importance to examine the role of moral values and belief in sacrificial dilemmas although we have just a little evidence about the role of ideological beliefs on utilitarian tendencies. Individuals higher on social dominance orientation and more likely to dehumanize others were more prone to utilitarian responses (Takamatsu, 2019). This last result is particularly interesting, as dehumanizing beliefs are conceptually close to one of Bandura’s moral disengagement mechanisms and, although no study has investigated so far the association between these mechanisms and utilitarian tendencies, there is empirical evidence that moral disengagement is positively associated with increased unethical decision-making (Detert et al., 2008; Johnson and Connelly, 2016).

Surprisingly, there is a lack of research investigating the role of daylife contexts on deontological vs. utilitarian judgments and most of the studies have been carried out with the adults’ population. Just a few studies, to our knowledge, took into consideration family variables evaluating the effects of adult attachment style (Koleva et al., 2014) and childhood adversity on adult moral decision-making (Larsen et al., 2019). Findings of these studies evidenced that avoidant attachment (Koleva et al., 2014), as well as higher levels of physical neglect during childhood (Larsen et al., 2019), were associated with greater acceptability of causing harm to utilitarian ends.

Finally, as regards the role of community context, to date, we are not aware of any study having investigated its association with moral judgment in sacrificial moral dilemmas. However, there is a great amount of evidence highlighting that growing up in violent contexts and being repeatedly exposed to the observation of violent models within the community makes youth desensitized to the effects of violence (Huesmann and Kirwil, 2007), thus disrupting their ability to empathize with other’s pain and suffering and making them more prone to condone harmful actions toward others.

### Utilitarian Vs. Deontological Choice in Developmental Age

Despite the wide literature on the mechanisms and psychological correlates of sacrificial dilemmas, very few studies have been carried out with non-adult participants. Research has therefore largely disregarded the developmental perspective, ignoring that adolescence is a critical period in the consolidation of personal values, in the formation of the moral self and identity, and, in general, for the development of cognitive capabilities which could reasonably influence moral choices. A possible reason is that in a nativist perspective (e.g., Mikhail, 2000), children and adults should not differ in making moral judgments that are undergone/subjected to the same mechanisms.

A study by Pellizzoni et al. (2010) seems to confirm this assumption. The authors proposed to 3-year-old children the switch and the footbridge dilemma using Lego constructions to adapt scenarios. They found similar patterns in utilitarian vs. deontological responses of children compared to adults. Both groups preferred utilitarian choice (benefit for the greater number of people) only if the required action did not imply a personal contact: 87% of children advocated action to save five lives in the trolley dilemma, against the 27% in the footbridge dilemma. These percentages were similar to those found in adults: 91% of subjects judged the action of hitting the switch as appropriate, whereas only 31% judged it as appropriate to push the man onto the tracks. The authors concluded that in some situations, fast and automatic intuitions, based on emotional arousal, are the primary source of many moral judgments and that deliberation is used mostly to construct post hoc justifications for judgments that have already occurred (Haidt, 2001).

In line with the literature evidencing an association between utilitarian responses to sacrificial dilemmas and psychopathic/antisocial tendencies (Kahane et al., 2015), two studies investigated this association in adolescents (Bacchini et al., 2018; Dickinson and Masclet, 2019) finding results consistent with those from studies conducted with adults. More specifically, Bacchini et al. (2018) found a higher tendency to make utilitarian choices in incarcerated adolescents compared to a community control group, and the mediating role of utilitarian choice in the relationship between perspective-taking and delinquent behavior.

A study with two groups of adolescents (9th- and 12th-grade students) was realized by Stey et al. (2013) to investigate whether judgments in sacrificial dilemmas were influenced by affective considerations (Greene et al., 2001) and whether judgments of permissible harm were the product of implicit principles (Cushman et al., 2006). The authors did not find age differences between younger and older adolescents in the frequency of utilitarian vs. deontological choices, even though 12th-grade students provided significantly more sufficient justifications than 9th-grade students when asked to justify their judgments. Their conclusions agreed with Greene’s point of view, since participants were more likely to use emotion words rather than refer to implicit principles, like contact and action, in their justifications, and this tendency to use emotion words in justifications was related to more deontological responses.

On the other hand, harsh criticism of Greene’s model inspired the research involving adolescents carried out by Dahl et al. (2018). In their study, the authors proposed a divergent interpretation of the trolley problem and its variants, contrasting the dichotomy (emotion vs. cognition) postulated by Greene. They argued that the moral reasoning about sacrificing and saving lives involve multiple moral considerations about the value of life, that cannot simply be considered post hoc rationalizations (Haidt, 2013). In line with the literature, also Dahl et al. (2018) found that 71% of adolescents judged it permissible to activate the switch, whereas only 19% judged it permissible to push the large man onto the tracks. Nevertheless, investigating qualitative differences in participants’ reasoning about the standard switch and footbridge situation, they found that in the switch dilemma people reasoned in a utilitarian way, whereas in the footbridge dilemma people’s reasoning was multifaceted, implying other morally relevant issues concerning the value of life (based on the number of saved lives or the right to life of the potential victim), the natural course of events, the responsibility for consequences of actions, and the evaluation of the consequences for self. In addition, consistently with Bleske-Rechek et al. (2010), they found that choices and justifications of adolescents and adults change accordingly to other variants added to the dilemmas (e.g., the man on the bridge will be only scratched but not dead, or the victim on the sidetrack was a relative of the observer). In conclusion, Dahl et al. (2018, p. 14) considered the trolley problem (in both variants) as a dilemma evoking multifaceted conflicts which individuals try to solve according to their moral beliefs. In this regard, they affirm that “changes in evaluations about multifaceted situations reflect developmental changes in how children coordinate competing moral and non-moral considerations.”

### The Present Study

An extensive amount of research on sacrificial dilemmas has been produced in the last two decades. However, only a few studies have been conducted with adolescents, although adolescence is a crucial developmental period for the consolidation of moral beliefs. The present study aims to fill this gap in the literature, investigating adolescents’ judgments in sacrificial dilemmas and the role of emotional-, contextual-, and moral-related variables.

The first research question was whether adolescents made more utilitarian vs. deontological choices in the switch dilemma compared to the footbridge dilemma. Consistently with the large number of studies carried out with adults and the few existing findings concerning adolescents, we expected more utilitarian responses to the switch dilemma and more deontological responses to the footbridge dilemma.

The second research question was whether adolescents’ responses varied by gender and school grade, used as a proxy of adolescent age. Previous studies evidenced a higher prevalence of deontological responses among females (Fumagalli et al., 2010; Bartels and Pizarro, 2011; Friesdorf et al., 2015; Capraro and Sippel, 2017; Armstrong et al., 2019). On the other hand, no study has systematically investigated differences between younger and older adolescents; therefore, we were not able to advance specific hypotheses concerning this issue.

The third research question was whether emotional- (i.e., callous-unemotional traits), contextual- (i.e., community violence exposure and parental rejection), and moral-related variables (i.e., moral disengagement and universalism value) could differently affect the tendency to give utilitarian vs. deontological responses across the two moral dilemmas. In this regard, according to the assumption of the dual-process theory, the effect of the aforementioned variables should be especially notable in the footbridge dilemma, given the higher emotional activation that this dilemma is expected to produce. More specifically, with respect to the callous-unemotional traits, we hypothesized that individuals with low levels of emotional activation as those with higher callous-unemotional traits were more likely to give utilitarian responses to the footbridge dilemma compared to the switch dilemma.

At the contextual level, we investigated the role of some negative experiences that adolescents could have encountered within their family context and in the neighborhood. Only two studies carried out with adults investigated the role of family-related dimensions on moral judgment in sacrificial moral dilemmas, finding that avoidant attachment (Koleva et al., 2014) and childhood adversity, such as physical neglect (Larsen et al., 2019), were associated with a higher frequency of utilitarian choices. Based on the findings of these studies, we hypothesized that higher levels of perceived parental rejection were associated with a higher tendency to give utilitarian responses in the footbridge dilemma, as parental rejection could affect emotional responsiveness in children and obstacle the internalization of moral values (Grusec et al., 2000). We also investigated the role of adolescents’ exposure to community violence. Although, to our knowledge, no study has so far investigated whether experiencing violence within the neighborhood/community could affect the moral decision-making in sacrificial moral dilemmas, previous research (e.g., Dodge et al., 2006) highlighted that growing up in a violent neighborhood/community might undermine the normative process of moral development. Therefore, we speculated that, due to a process of desensitization to violence resulting from repeated experiences of exposure to community violence (Huesmann and Kirwil, 2007), youth could reduce their emotional aversion to performing even “up-close and personal” harm to others, as in the case of footbridge dilemma. Lastly, we investigated the role of two moral-related variables by considering the contribution of moral disengagement and the value of universalism. As a utilitarian solution in sacrificial moral dilemmas requires that individuals come to consider acceptable harming others for the sake of a greater good, and moral disengagement mechanisms are defined as leading individuals to disengage moral self-sanctions from their harmful practices, it is plausible to hypothesize that higher levels of moral disengagement could be associated with a higher prevalence of utilitarian responses. Moreover, since utilitarian responses to the footbridge dilemma require more sophisticated reasoning to justify the choice of sacrificing one life through direct action, we hypothesized that the more youth make use of moral disengagement mechanisms to justify their actions, the more they tend to make utilitarian choices in the footbridge dilemma.

Regarding the role of values, despite the lack of studies investigating their link with moral decision-making in sacrificial dilemmas, it seems reasonable to assume that, among others, the value of universalism which focused on the importance of preserving human life, could encourage the adoption of a deontological rather than utilitarian perspective in sacrificial moral dilemmas.

## Materials and Methods

### Participants and Procedure

The sample consisted of 755 Italian adolescents (54.7% females) enrolled in grade 10 (*n*=459, 60.8%; *M*_age_=15.25, *SD*=0.63) and grade 13 (*n*=296, 39.2%; *M*_age_=18.27, *SD=*0.63) of several public schools located in the metropolitan area of Naples. The mean age of the total sample was 16.45 (*SD=*1.61), ranging from 14 to 20years. Although the mean age of students enrolled in the grade 13 is very close to the age of undergraduates participating in other studies, in Italy these subjects still involved in their high school careers, are usually considered representative of the adolescent age group. The socioeconomic distribution of participants’ families reflected the Italian national statistics [Istituto Nazionale di Statistica (ISTAT), 2018], with most of the fathers and mothers having obtained at least a high school degree (30.9% of fathers and 34.3% of mothers) or a junior high school license (55.8% of fathers and 51.1% of mothers).

The study was approved by the Ethical Committee of the Department of Humanistic Studies, University of Naples Federico II (project identification code: 2/2020). Data were collected by trained research assistants in 2017, during regular school hours. Parents’, or child guardians’ written informed consent and adolescents’ assents were obtained before the administration of the questionnaires. Privacy was guaranteed to participants in accordance with Italian laws 196/2003 and 101/2018. Participation in the study was voluntary, and participants could withdraw at any time without any adverse consequence.

### Measures

#### Moral Dilemmas

Participants were presented with two scenarios involving hypothetical moral dilemmas extracted from Greene et al. (2009) and Paxton et al. (2012). The problem was presented as follows: A runaway trolley is about to run over and kill five people. In the “switch” scenario (impersonal sacrificial dilemma), one can save them by hitting a switch that will divert the trolley onto a sidetrack, where it will kill only one person. In the “footbridge” scenario (personal sacrificial dilemma), one can save them by pushing a large man off a footbridge and onto the trolley’s path, killing him, but stopping the trolley. Following Greene et al. (2001) and Paxton et al. (2012) for each scenario, participants had to indicate whether the proposed action was “morally acceptable” or not. Choosing “no” (i.e., it is not morally acceptable switching tracks, or pushing the person off the bridge) can be classified as a deontological moral judgment. Choosing “yes” (i.e., it is morally acceptable switching tracks, or pushing the person off the bridge) can be classified as a utilitarian moral judgment. No/Yes answers were used in the analyses as a dichotomous dependent variable.

#### Moral Disengagement

Moral disengagement was measured through the moral disengagement scale developed by Caprara et al. (2006). The questionnaire specifically assesses the proneness to morally disengage with reference to different forms of detrimental conduct, in different contexts and interpersonal relationships. It consisted of 24 items that participants rated on a 5-point Likert-type scale (from 1 = “agree not at all” to 5 = “completely agree”). Sample items were as follows: “If people leave their belongings around, it is their fault if someone steals them” and “People cannot be held responsible for crimes committed at the instigation of others.” Cronbach’s alpha and McDonald’s omega were 0.95.

#### Universalism

Value of universalism was self-reported by participants using the Portrait Values Questionnaire – short version (PVQ; Schwartz et al., 2001; Italian validation by Capanna et al., 2005). The PVQ – short version includes 21 verbal portraits of different people that describe a person’s goals, aspirations or wishes, and point implicitly to the importance that the person attaches to a specific value. For each portrait, respondents answered the question “How much like you is this person”? using a 6-point Likert scale (from 1=“not at all like me” to 6=“very much like me”). For the purposes of this study, only the items measuring universalism were considered (3 items). A sample item was “He/she thinks it is important that every person in the world should be treated equally.” Cronbach’s alpha and McDonald’s omega were 0.62.

#### Callous-Unemotional Traits

Callous-unemotional traits were measured using the 24-item Inventory of Callous-Unemotional Traits (ICU; Kimonis et al., 2008; Italian validation by Ciucci et al., 2014). Items of the questionnaire were scored along a 4-point Likert-type scale (from 0=“not at all true” to 3=“definitely true”). The factor structure of the ICU, as it has been demonstrated in several previous studies (e.g., Kimonis et al., 2008; Roose et al., 2010; Ciucci et al., 2014), consists of a general callous-unemotional factor and three subfactors: callousness (e.g., “The feelings of others are unimportant to me”), unemotional (e.g., “I hide my feelings from others”), and uncaring (e.g., “I try not to hurt others’ feelings” – reversed scored item). For this study’s purposes, items were averaged and used as a general callous-unemotional factor. Cronbach’s alpha and McDonald’s omega for the global scale were 0.81 and 0.80, respectively.

#### Parental Rejection

The Parental Acceptance-Rejection Questionnaire (PARQ; Rohner et al., 2005) was used to measure adolescents’ perceptions of maternal and paternal rejection. Participants completed the mother version of the PARQ and then the father version. The PARQ is a 24-item self-report instrument that assesses respondents’ perceptions of parental warmth, affection, hostility, aggression, indifference, neglect, and undifferentiated rejection. Items were rated on a 4-point Likert scale (from 1=“almost never true” to 4=“almost always true”). Sample items were as follows: “My [mother/father] makes me feel wanted and needed”; “My [mother/father] goes out of [her/his] way to hurt my feelings.” Scores for each subscale were averaged to compute global scores of maternal and paternal rejection, with high values indicating high rejection. The two scores, one referring to the mother and the other one referring to the father, were then averaged to create a composite score of parental rejection, which demonstrated high reliability (Cronbach’s alpha=0.95; McDonald’s omega=0.94).

#### Exposure to Community Violence

Exposure to community violence was assessed using the witnessing subscale of the Exposure to Community Violence Questionnaire (Esposito et al., 2017), consisting of 6 items. Adolescents were asked to report the frequency with which they have been witnessed violent incidents that had occurred during the last year in their neighborhood using a scale ranging from 1 (“never”) to 5 (“more than five times”). A sample item was “How many times have you seen somebody get robbed”? Scores for each item were averaged to create the score for community violence exposure. Reliability statistics were adequate, Cronbach’s alpha and McDonald’s omega=0.88.

### Statistical Analysis

Before testing our hypotheses, the univariate normality of data distribution was tested, finding that no study’s variables approached skewness > |3| or kurtosis > |10|.

Then, we firstly identified within the sample participants who made deontological vs. utilitarian judgments to impersonal (i.e., switch dilemma) and personal (i.e., footbridge dilemma) moral scenarios (Research question 1) and compared them by gender (males vs. females) and school grade (10th vs. 13th graders; Research question 2) using a set of chi-square statistics performed in IBM SPSS 21 (IBM, Armonk, NY, United States).

The third research question (Research question 3, i.e., correlates of moral choices) was then examined using generalized linear mixed models with binomial family and logit link function, performed with JASP statistical software (JASP Team, 2020). The dependent variable was a dichotomous variable indicating whether the action required in the dilemmas was considered morally acceptable or not (no vs. yes). Models included random intercepts for participants and fixed effects of the variables considered in the study as potential predictors. More specifically, three separate generalized linear mixed models were performed. The first one tested the effects of emotional traits (namely, callous-unemotional traits), controlling for gender, school grade, and type of moral scenario (switch vs. footbridge). Then, the two-way interaction between emotional traits and type of scenario was included as a second step. The second and third models examined the effects of contextual factors (exposure to community violence and parental rejection) and moral-related variables (moral disengagement and universalism), respectively. Also in these cases, the effects of gender and school grade were controlled, and interactions with the moral scenario were tested. Continuous variables were mean-centered before running the analyses. Model terms were tested with the likelihood ratio tests method.

## Results

### Deontological Vs. Utilitarian Responses: Differences by Type of Scenario, Gender, and School Grade

In order to examine whether adolescents’ judgments differed according to the type (personal vs. impersonal) of moral scenarios (Research question 1) and whether there were gender- and school grade-related differences (Research question 2), a set of chi-square statistics was performed. Results revealed that there were significant differences [*χ*^2^ (1)=84.31; *p*<0.001] in the frequency of deontological (vs. utilitarian) responses to the switch dilemma (268 subjects; 35.5%; *M*=109, *M*_age_=16.60years, *SD=*1.56) compared to the footbridge dilemma (543 subjects; 71.9%; *M*=221, *M*_age_=16.49years, *SD=*1.60). Moreover, significant differences by gender [*χ*^2^ (1)=16.50; *p*<0.001] emerged only when youth were faced with the footbridge dilemma, with 121 males out of 342 (35.4%) making utilitarian judgments compared to 91 females out of 413 (22%). Conversely, no significant gender difference emerged when subjects were presented with the switch dilemma, with 233 males out of 342 (68.1%) making utilitarian judgments compared to 254 females out of 413 (61.5%). Finally, significant school grade differences emerged when youth were faced with both the switch [*χ*^2^ (1) = 4.06; *p*<0.05] and the footbridge dilemma [*χ*^2^ (1) = 4.73; *p*<0.05], with 10th-grade participants more likely to make utilitarian judgments than 13th-grade participants (67.3% vs. 60.1 and 30.9% vs. 23.6%, for switch and footbridge dilemma, respectively).

### Generalized Linear Mixed Models

The results of the generalized linear mixed models (Research question 3) are displayed in Tables 1–3 and described in the following sections.

**Table 1:** Generalized linear mixed model (1) – Emotional traits predicting No/Yes answers in moral dilemmas.

Terms	*B*	*SE*	*t*	*p*
Intercept	−0.27	0.09	−2.84	0.005
Type of moral scenario (Switch)	1.18	0.10	11.98	<0.001
Gender (Male)	0.30	0.09	3.22	0.001
School Grade (10th)	0.20	0.09	2.11	0.035
Callous-unemotional traits	0.28	0.22	1.27	0.203
**Model with interactions**				
Intercept	−0.28	0.10	−2.91	0.004
Type of moral scenario (Switch)	1.21	0.10	11.85	<0.001
Gender (Male)	0.31	0.10	3.22	0.001
School Grade (10th)	0.20	0.10	2.14	0.033
Callous-unemotional traits	0.31	0.22	1.37	0.170
Type of moral scenario * Callous-unemotional traits	−0.44	0.17	−2.57	0.010

**Table 2:** Generalized linear mixed model (2) – Contextual-related factors predicting No/Yes answers in moral dilemmas.

Terms	*B*	*SE*	*t*	*p*
Intercept	−0.27	0.09	−2.89	0.004
Type of moral scenario (Switch)	1.18	0.10	11.98	<0.001
Gender (Male)	0.31	0.09	3.32	<0.001
School Grade (10th)	0.22	0.09	2.37	0.018
Parental rejection	0.38	0.18	2.07	0.039
Community violence	−0.12	0.11	−1.10	0.273
**Model with interactions**				
Intercept	−0.32	0.10	−3.25	0.001
Type of moral scenario (Switch)	1.20	0.10	11.70	<0.001
Gender (Male)	0.31	0.10	3.26	0.001
School Grade (10th)	0.22	0.10	2.34	0.019
Parental rejection	0.32	0.18	1.73	0.085
Community violence	−0.10	0.11	−0.86	0.393
Type of moral scenario * Parental rejection	−0.35	0.14	−2.46	0.014
Type of moral scenario * Community violence	−0.21	0.08	−2.56	0.010

**Table 3:** Generalized linear mixed model (3) – Moral-related variables predicting No/Yes answers in moral dilemmas.

Terms	*B*	*SE*	*t*	*p*
Intercept	−0.27	0.09	−2.83	0.005
Type of moral scenario (Switch)	1.18	0.10	11.98	<0.001
Gender (Male)	0.28	0.10	2.93	0.003
School Grade (10th)	0.19	0.10	1.99	0.046
Moral disengagement	0.16	0.13	1.28	0.200
Universalism value	−0.08	0.09	−0.82	0.412
**Model with interactions**				
Intercept	−0.30	0.10	−3.01	0.003
Type of moral scenario (Switch)	1.26	0.11	11.52	<0.001
Gender (Male)	0.30	0.10	2.93	0.003
School Grade (10th)	0.21	0.10	2.08	0.037
Moral disengagement	0.18	0.13	1.37	0.172
Universalism value	−0.09	0.10	−0.92	0.356
Type of moral scenario * Moral disengagement	−0.38	0.10	−3.81	<0.001
Type of moral scenario * Universalism value	0.17	0.07	2.30	0.021

#### The Effect of Emotional Traits

**Figure 1:**
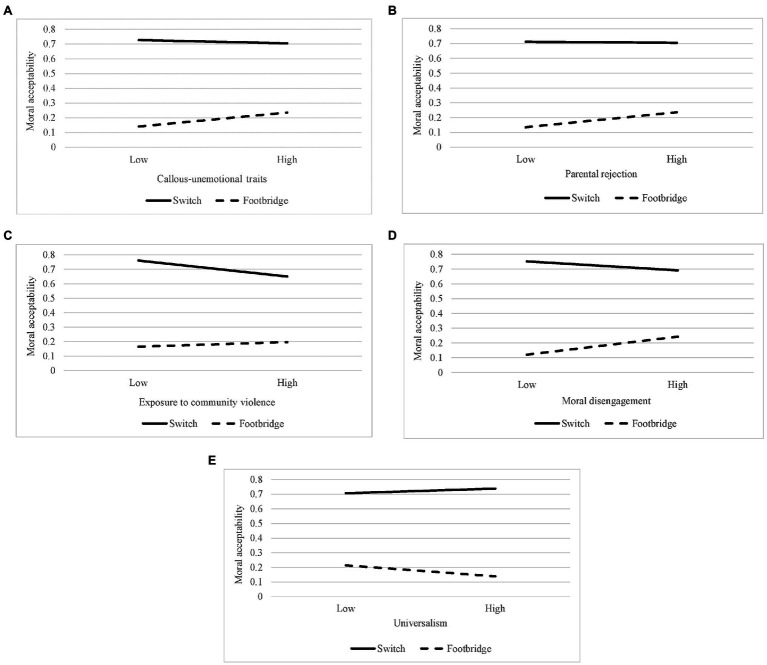
Plots of the effects of emotional traits **(A)**, context-related factors **(B, C)**, and moral-related variables **(D, E)** depending on the type of moral scenario (switch vs. footbridge).

The results showed a significant interaction effect between callous-unemotional traits and the type of moral dilemma (Table 1). The analysis of simple slopes (Figure 1A) indicated that those who reported higher levels of callous-unemotional traits were more likely to rate the intervention in the footbridge scenario as permissible, *B*=0.75, *SE*=0.29, 95% CI [0.18, 1.32], whereas no significant effect was found in the switch situation, *B*=−0.14, *SE*=0.27, 95% CI [−0.67, 0.39].

#### The Effects of Contextual Factors

The examination of the effects of parental rejection and community violence witnessing revealed significant interaction effects with the type of moral dilemma (Table 2). More specifically, parental rejection was found to have a significant positive effect only in the footbridge scenario, *B*=0.66, *SE* = 0.24, 95% CI [0.20, 1.12], whereas no significant effect emerged in the switch scenario, *B*=−0.03, *SE* = 0.23, 95% CI [−0.48, 0.41] (Figure 1B). Conversely, high levels of exposure to community violence negatively predicted the ratings of the intervention as permissible in the switch situation, *B*=−0.31, *SE* = 0.14, 95% CI [−0.58, −0.04], whereas no significant effect was found in the footbridge scenario, *B*=0.12, *SE* = 0.14, 95% CI [−0.16, 0.39] (Figure 1C).

#### The Effects of Moral-Related Variables

Moral disengagement and universalism were found to be significant predictors of participants’ ratings of the intervention as permissible, conditional on the type of moral dilemma (interaction effects: *p*s<0.001 and 0.05, for moral disengagement and universalism, respectively; see Table 3). The simple slopes analysis revealed that both had a significant effect only in the footbridge situation. Moral disengagement was associated with an increased likelihood to rate the intervention as permissible, *B*=0.57, *SE* = 0.17, 95% CI [0.23, 0.90] (Figure 1D), whereas high levels of universalism negatively predicted the permissibility of the intervention, *B*=−0.26, *SE* = 0.12, 95% CI [−0.50, −0.01] (Figure 1E).

## Discussion

In the last two decades, a tremendous amount of research has been carried out using the “trolley dilemma” in which participants are faced with the choice of whether or not it is permissible to sacrifice one human life to save five others. Such sacrificial dilemma (and its variants, e.g., the footbridge dilemma) has become a prototypical tool of investigation because it would allow to reveal two competing mechanisms implicated in making moral judgments: a cognitive-driven process (addressing utilitarian choices, i.e., based on an evaluation of cost-benefit ratio, it is morally acceptable to kill one person in order to save five others) as opposed to an emotion-driven process (addressing deontological choices, i.e., based on an immediate, automatic and unconscious feeling that it is not morally acceptable to kill one person in order to save five others). According to the dual-process theory of moral judgment (Greene et al., 2001; Greene, 2007), while utilitarian choices are activated when individuals are faced with a low-impact dilemma (i.e., “impersonal” trolley dilemma) that elicits lower levels of emotional distress, deontological choices are activated when the proposed high-impact dilemma (i.e., “personal” footbridge dilemma) elicits higher levels of emotional distress.

The present study aimed to increase the knowledge about the processes underlying moral decision-making into sacrificial dilemmas in two ways: (1) by exploring the adolescents’ responses to trolley dilemma across different, “personal” (i.e., the switch scenario) and “impersonal” (i.e., the footbridge scenario) variants since, to date, only a few studies (e.g., Dahl et al., 2018) on this topic have involved non-adults populations; (2) by investigating the concurrent contribution of gender, age, emotional-, contextual- (i.e., family and neighborhood), and moral-related variables in making moral judgments (deontological vs. utilitarian) in both switch and footbridge dilemmas.

Consistent with the literature, in our study, we found that adolescents’ choices in sacrificial moral dilemmas significantly varied according to the type (impersonal vs. personal) of scenario, with the majority of youths more prone to the utilitarian choice in the switch dilemma and, on the contrary, more likely to choose the deontological solution in the footbridge dilemma (Research question 1). These findings are in line with our expectations and with previous studies showing similar patterns of responses allowing harm to others to utilitarian ends more in impersonal than in personal dilemmas, among 3-year-old children (Pellizzoni et al., 2010), adolescents (Dahl et al., 2018), and adults from different countries (Awad et al., 2020). Moreover, we found that younger participants were more prone to utilitarian responses, irrespective of the type of dilemma, and that there were gender differences depending on the type of dilemma, with males more willing to choose utilitarian solutions only in footbridge dilemma (Research question 2). These results are consistent with those emerged in previous studies in which males showed a stronger preference for utilitarian over deontological judgments (e.g., Fumagalli et al., 2010; Bartels and Pizarro, 2011; Friesdorf et al., 2015), particularly when considering “personal” moral dilemmas (Fumagalli et al., 2010) where harm requires physical force (Greene et al., 2009). Moreover, our results seem to make sense considering the wide research highlighting higher emotional responsiveness among females (Eisenberg, 2005), which could lead to giving more automatic and immediate responses evidencing aversion to causing harm to others in the context of moral dilemmas. On the other hand, males’ moral evaluation is believed to be more pragmatic and adhering to abstract principles of justice (Jaffee and Hyde, 2000; see also the classical debate Kohlberg vs. Gilligan), although a recent meta-analysis (Friesdorf et al., 2015) suggested that gender differences in the preferences for utilitarian vs. deontological judgments stem from gender differences in affective reactions to causing harm rather than in cognitive evaluations of outcomes. Results regarding age-related differences are something new, as the present study is, to our knowledge, the first one to systematically investigate differences between younger and older adolescents with respect to moral judgments in sacrificial moral dilemmas. The finding that younger adolescents are more prone to the utilitarian solution, irrespective of the type of dilemma would suggest that the preference for the deontological vs. utilitarian solution has more to do with developmental changes than with the characteristics of the proposed scenario. However, younger adolescents tend to make, as well as older adolescents, more utilitarian choices in the switch dilemma than in the footbridge dilemma, in accordance with the expectation of Greene’s dual-process model.

With respect to our third research question, which concerned the investigation of moral choices’ correlates, we performed a set of generalized linear mixed models in order to test the contribution of emotional- (i.e., callous-unemotional traits), contextual- (i.e., parental rejection and community violence witnessing), and moral-related (i.e., moral disengagement and universalism) variables in making moral judgments (i.e., deontological vs. utilitarian) in sacrificial dilemmas. Both the main effects of each variable (i.e., without take into account the type of dilemmas) and the interaction effects (i.e., testing whether the contribution of each variable varied depending on the type of dilemmas) were tested. Our first result was that adolescents higher on callous-unemotional trait were more likely to choose the utilitarian solution (i.e., push the large man onto the tracks to save five other people) in the footbridge case, while we did not find any difference in the switch case. This finding was in line with our hypotheses and with the literature evidencing that clinical psychopaths (Koenigs et al., 2012; Rosas and Koenigs, 2014), as well as subclinical individuals with psychopathic tendencies, (Glenn et al., 2010; Bartels and Pizarro, 2011; Langdon and Delmas, 2012; Gao and Tang, 2013; Djeriouat and Trémolière, 2014; Kahane et al., 2015; Patil, 2015; Balash and Falkenbach, 2018) are more willing to accept utilitarian solution when facing emotionally aversive moral dilemmas. Callous-unemotional traits, which can be considered the hallmark of the psychopathic personality (Blair, 2013), are characterized, indeed, by general disregard for others, lack of empathy and, more in general, deficient emotional activation. Therefore, it is not surprising that individuals higher on these personality traits are less responsive to the moral salience of a personal moral dilemma, as the footbridge case. Moreover, this conclusion is in line with studies evidencing a higher tendency to make utilitarian choices in subjects with low levels of affective empathy (Koleva et al., 2014; Patil and Silani, 2014; Takamatsu and Takai, 2019; Dinić et al., 2021) or in a clinical population with brain injuries altering affective reactions (see Greene, 2014).

Then, we took into consideration contextual factors, investigating the role of two negative experiences that adolescents could have encountered within their family context and in the neighborhood: perceived parental rejection and exposure to community violence as a witness. In the present study, the two contextual dimensions showed divergent interactions with the type of dilemma. Indeed, adolescents perceiving higher parental rejection are more prone to the utilitarian choice in the footbridge dilemma, while we did not find any difference in the switch dilemma. Conversely, adolescents who are more often witnesses of violence in their neighborhood are less prone to the utilitarian solution in the switch scenario, while we did not find any difference in the footbridge situation. Although only two studies involving adults and focusing on different variables (attachment style and childhood adversity, such as physical neglect during childhood) investigated the role of family-related dimensions on moral judgment in sacrificial moral dilemmas (Koleva et al., 2014; Larsen et al., 2019), their results evidenced that dysfunctional relationships (avoidant attachment and higher physical neglect) within the family context can promote utilitarian tendencies. Moreover, there is evidence in the literature that parental rejection affects emotional responsiveness in children and hinder the normal process of internalization of moral values (Grusec et al., 2000), leading to various maladaptive outcomes including internalizing and externalizing symptoms. Therefore, parental rejection could work, in line with the basic assumption of Greene’s theory, as another variable influencing, at a contextual level, the emotional and cognitive processes involved in moral decision-making. Moreover, the reduced moral responsiveness could at least in part explain why we found differences only when adolescents considered the footbridge case. With respect to exposure to community violence, to our knowledge, there is no study in the literature evaluating the association of this contextual variable with utilitarian vs. deontological choice in sacrificial moral dilemmas. However, there is a great amount of evidence highlighting the negative effects of community violence exposure on moral development. The research found that children and adolescents exposed to community violence, fail in distinguishing moral vs. conventional issues (Bacchini et al., 2013), tend to make frequent recourse to self-serving cognitive distortions (Dragone et al., 2020; Esposito et al., 2020), judge morally acceptable physically harming others in contexts of survival or revenge (Posada and Wainryb, 2008), and are more likely to condone moral transgressions when provoked or for reasons of retaliation (Ardila-Rey et al., 2009). Overall, exposure to violence has been found to substantially disrupt the moral decision-making ability as a result of impairments of several emotional (e.g., empathy), cognitive (e.g., theory of mind), and inhibitory control abilities (Zucchelli and Ugazio, 2019).

Growing up in violent communities could exert a detrimental effect on normative moral development, leading to a decreased sensitivity toward violence (Dodge et al., 2006; Huesmann and Kirwil, 2007). Therefore, harmful behaviors end up becoming normative and could also result in a sort of indifference with other’s pain and suffering and, at the same time, a sort of learned helplessness that induces individuals not to interfere with the natural course of events, just like our adolescents.

Finally, we evaluated the possible role of two moral-related variables: moral disengagement and universalism, the basic human value representing the intrinsically moral goal of preserving the welfare of others. Our results showed that adolescents more prone to make use of moral disengagement mechanisms were more likely to choose utilitarian solutions in the footbridge situation, while we did not find any difference in the switch situation. Conversely, adolescents higher on universalism proved to give fewer utilitarian responses in the footbridge case, while no difference emerged in the switch case.

Although there is no study, to our knowledge, in the literature investigating the role of moral disengagement with respect to the tendency to give utilitarian vs. deontological responses in sacrificial moral dilemmas, there is little evidence of the role of beliefs in making moral judgments. In particular, as shown by Takamatsu (2019), individuals higher on social dominance orientation and, even more interesting for the present study, individuals more likely to dehumanize others were more prone to utilitarian responses. As dehumanizing beliefs are conceptually close to one of Bandura’s moral disengagement mechanisms, this evidence in the literature seems to support our results. Moreover, our findings seem to make sense, considering that moral disengagement mechanisms are defined as allowing individuals to disengage moral self-sanctions from their harmful practices and utilitarian choice in sacrificial moral dilemmas, in particular in personal scenarios such as the footbridge case, requires individuals to consider acceptable harming others for the sake of a greater good. With respect to the role of universalism, although there is no study in the literature investigating the association with moral decision-making in sacrificial dilemmas, it seems to make sense that a higher endorsement of values focused on preserving human life could lead to a higher tendency to deontological choices, in particular in personal dilemmas, in which the higher moral salience of the proposed scenario makes more difficult for individuals to set aside their values. Moreover, our result receives support from the studies evidencing an association between a reduced tendency to prefer utilitarian solutions in sacrificial moral dilemmas and a higher endorsement of moral foundations, in particular of the Care/Harm foundation, underlying aversion to harmful actions (Djeriouat and Trémolière, 2014; Koleva et al., 2014; Crone and Laham, 2015) and conceptually close to universalism. Finally, consistent with our findings, Dahl et al. (2018), investigating the qualitative differences in participants’ reasoning about the switch and the footbridge situation, found that unlike what happens in the switch case, in the footbridge case reasoning was more multifaceted, involving different moral considerations primarily associated with the value of life.

### Strengths, Limitations, and Future Perspectives

One of the major strengths of the present study is the focus on adolescence, largely disregarded in the research investigating so far moral decision-making in sacrificial moral dilemmas, despite its critical role for moral development. Focusing on adolescents, moreover comparing two age cohorts, allow us to understand utilitarian vs. deontological choices from a developmental perspective, evidencing whether and how moral judgments change over time. Furthermore, investigating the role of a broad range of possible correlates at the individual and contextual level and allows us to get a clearer picture of which features are more relevant with respect to the development of utilitarian vs. deontological inclinations.

On the other hand, our understanding of the developmental aspects of moral decision-making in sacrificial dilemmas is limited by the cross-sectional nature of the study, not allowing us to determine causal influences. More research involving longitudinal samples is needed to confirm and deepen our results. Another limitation is related to the use of sacrificial dilemmas as a measure of utilitarian vs. deontological inclination. Such measure is the subject of an ongoing scientific debate discussing the idea that this methodology has different weaknesses, such as treating utilitarian and deontological responses as inversely related (Conway and Gawronski, 2013), lack of manipulation of consequences and norms, that are the defining aspects of utilitarianism and deontology (see Gawronski and Beer, 2017), and directly measuring only a negative dimension of utilitarianism, called “instrumental harm” (Kahane et al., 2018). However, despite the proposal of adapting the traditional dilemma methodology (Conway and Gawronski, 2013; Gawronski et al., 2017) or replacing it with new measures (Kahane et al., 2018), the use of sacrificial moral dilemmas remains widely accepted (see Conway et al., 2018; Kahane et al., 2018), with the recommendation of taking in mind the limitations that were evidenced and that should be therefore extended to our results. In particular, our findings evidencing adolescents higher on different variables associated with maladaptation (higher callousness, more parental rejection) are more willing to sacrifice the man in the footbridge situation and seem to suggest that utilitarian choices are more likely to stem from a decreased aversion to harming others, rather than reflect a genuine concern for the greater good. Accordingly, the finding that younger adolescents tend to give more utilitarian responses, irrespective to the proposed scenario, could be read as in part reflecting the incomplete maturation of nervous system, resulting in a still incomplete development of executive. functions However, the criticisms we have mentioned above require caution, in view of the difficulty to ascertain the underlying mechanism of the adolescents’ choices. More research is needed to shed light on this issue.

Moreover, future research should clarify how aversive experiences within the family or community exert their influence on adolescents’ moral decision-making in sacrificial dilemmas and, even more basically, which are the main characteristics of the adolescents choosing a deontological solution in the switch case. It would be also interesting deepen the role of cognitive variables, since previous research has evidenced that the reversal of moral preferences that can be observed when individuals face different moral scenarios, as in the switch and footbridge case, may occur because utilitarian moral judgments are cognitively too demanding (Da Silva et al., 2016). Finally, future studies could be useful to deepen the practical implications of results regarding the utilitarian vs. deontological choices and to explore the possible use of sacrificial dilemma as a tool to increase moral skills (Seider, 2009).

## Data Availability Statement

The datasets for this study can be found in the Supplementary Material.

## Ethics Statement

The study was reviewed and approved by the Ethical Committee of the Department of Humanties, University of Naples “Federico II” (project identification code: 2/2020). Written informed consent to participate in this study was provided by the participants’ parents, or legal guardian/next of kin.

## Author Contributions

DB, GDA, and MD contributed to conception and design of the study. GDA organized the database. GDA, MD, and CE performed the statistical analysis and wrote sections of the manuscript. DB wrote the first draft of the manuscript. All authors contributed to manuscript revision, read, and approved the submitted version.

## Conflict of Interest

The authors declare that the research was conducted in the absence of any commercial or financial relationships that could be construed as a potential conflict of interest.

## Publisher’s Note

All claims expressed in this article are solely those of the authors and do not necessarily represent those of their affiliated organizations, or those of the publisher, the editors and the reviewers. Any product that may be evaluated in this article, or claim that may be made by its manufacturer, is not guaranteed or endorsed by the publisher.
